# Comparison of Cycloplegic and Non-Cycloplegic Refraction in School-Aged Children: A Cross-Sectional, Observational Study

**DOI:** 10.7759/cureus.92899

**Published:** 2025-09-22

**Authors:** Tilottama Kar, Ankur K Shrivastava

**Affiliations:** 1 Ophthalmology, All India Institute of Medical Sciences, Patna, Patna, IND; 2 Ophthalmology, All India Institute of Medical Sciences, Raipur, Raipur, IND

**Keywords:** autorefraction, emmetropia, hypermetropia, myopia, spherical equivalent

## Abstract

Aim

The aim of the study was to evaluate the differences between cycloplegic and non-cycloplegic refraction in school-aged children.

Materials and methods

This cross-sectional, observational study was conducted at a tertiary care institute in central India, comprising 110 children aged 5-15 years. Visual acuity was recorded, and anterior segment examination was performed. Undilated autorefraction was done using an autokerato-refractometer. The participants were cyclopleged using eye drop cyclopentolate 1%, one drop every 15 minutes in both eyes, for over one hour. The vertical pupillary diameter was measured using slit lamp biomicroscopy. Cycloplegic autorefraction was done using the same autokerato-refractometer. Autorefraction values of the right eye were recorded to eliminate bias.

Results

The mean spherical equivalent (SE) in dioptres without cycloplegia was -0.54, and with cycloplegia was +0.18. The mean ± SD of the difference between cycloplegic and non-cycloplegic refraction was 0.72 ± 0.72. Without cycloplegia, SE determined 69 (62.7%) participants as myopic, six (5.5%) as emmetropic, and 35 (31.8%) as hypermetropic. Whereas, with cycloplegia, SE determined 45 (40.9%) as myopic, two (1.8%) as emmetropic, and 63 (57.3%) as hypermetropic.

Conclusion

The study discovered a strong correlation between SE (with cycloplegic) and SE (without cycloplegic), and this correlation was statistically significant. Myopia was overestimated, and hypermetropia was underestimated with non-cycloplegic refraction compared to cycloplegic refraction. Hence, cycloplegic refraction is recommended for the precise measurement of refractive error in children.

## Introduction

Vision, the most influential of our senses, holds significant importance in all aspects and phases of our lives. Vision impairment and blindness in children are major public health concerns worldwide [1]. Uncorrected refractive error is the most common reason why children experience visual impairment [2]. The World Health Organization (WHO) estimates that 19 million children and teenagers between the ages of five and 15 years suffer from vision impairment. Roughly 12.8 million (67%) of these instances have uncorrected refractive errors [3]. Uncorrected refractive errors can reduce productivity, educational possibilities, and life quality in general [4]. Therefore, early detection and appropriate management are essential.

A systematic review published in 2018 stated that the prevalence of myopia and hyperopia in India was 5.3% and 4%, respectively [5]. Children with untreated refractive errors develop strabismus and amblyopia. Furthermore, several vision-threatening ocular disorders, including glaucoma, myopic maculopathy, and retinal detachment, are associated with high myopia [6]. Therefore, it's critical to identify uncorrected refractive errors through comprehensive school vision screening programs, spread awareness, and create public health guidelines.

The eye's ability to bend incoming light rays is due to its refractive power, which is the combined power of the cornea and the lens. Furthermore, the accommodative power, which is defined as the ability of the ciliary body to change the lens curvature, also plays a role. The total increase in plus power caused by accommodation is called the amplitude of accommodation. Children often need cycloplegic refraction due to their high amplitude of accommodation. Cycloplegia blocks the action of the ciliary muscles, allowing for the measurement of only the objective component of refractive error. Cycloplegic eye drops are the most effective way to achieve paralysis of the ciliary muscles [7]. It is important to perform cycloplegic refraction because non-cycloplegic refraction can result in an incorrect classification of refractive error in children and adolescents [8].

The accommodative response in young phakic patients leads to an overestimation of myopia and an underestimation of hyperopia while conducting refraction without cycloplegia [9]. For this reason, cycloplegic refraction is regarded as the most reliable method for evaluating refractive errors in epidemiologic research involving children and adolescents [10]. With this background, the present study aimed to compare cycloplegic and non-cycloplegic refraction in school-aged children in Central India.

## Materials and methods

Study design and setting

This cross-sectional, observational study was conducted at the Department of Ophthalmology, All India Institute of Medical Sciences, Raipur, Chhattisgarh, India. The recruitment of patients was done from December 2022 to August 2023. The study met the Strengthening the Reporting of Observational Studies in Epidemiology (STROBE) guidelines. The study protocol was approved by the Institute Ethics Committee, All India Institute of Medical Sciences, Raipur, India (approval number: AIIMSRPR/IEC/2022/1260), and the tenets of the Declaration of Helsinki were strictly adhered to throughout the study. Written informed consent was obtained from the participants' guardians.

Study population

All children in the age group of 5-15 years who presented to the hospital with refractive errors were eligible for inclusion in the study. Participants with a history of any ocular comorbidities (conjunctivitis, uveitis, corneal opacities, corneal ulcer, glaucoma, nystagmus, and poorly fixating eye), adnexal comorbidities (acute or chronic dacryocystitis, chalazion, and stye), and a vertical pupillary diameter < 4 mm at the end of one hour of cycloplegia were excluded from the study. Participants with a history of any previous intraocular surgery (cataract or refractive surgery), extraocular surgery, or ocular trauma (open and closed globe injuries) were also excluded from the study. 

Sample size

The study size was calculated using a 95% confidence interval (CI) and 80% power. The standard deviation (SD) and minimum difference (d) detected for spherical value taken from a previous study [8], where mean + SD before cycloplegic refraction was -2.32+2.02 and after cycloplegic refraction was -1.78+2.03. The formula for sample size calculation -

$N = \frac{(Z_{\alpha} + Z_{\beta})^{2} \times \sigma^{2}}{\delta^{2}}$

Where N = sample size, Z_α_=1.96 at 95%CI, Z_β_= 0.84 at 80% power, σ= Combined SD (2.02+2.03/2=2.025), δ = difference between mean (=0.54)

Substituting the values in the formula, we got a total sample size of 110. After applying the eligibility criteria, we got a sample size of 130 children, which we kept to make up for any drop-out or loss due to non-achievement of the required pupillary dilatation.

Methodology

The participants underwent a comprehensive eye examination. Undilated autorefraction was done using an autokerato-refractometer (KR-1 Auto Kerato-Refractometer; Topcon Healthcare Solutions, Inc., New Jersey, United States). Participants were cyclopleged using eye drop cyclopentolate 1%, one drop every 15 minutes in both eyes, for over one hour. The vertical pupillary diameter was measured with a 1 mm wide slit beam using retro illumination in a slit lamp. Dilated autorefraction was done using the same autokerato-refractometer. The readings of undilated and dilated autorefraction of the right eye were recorded to eliminate bias in the study. Any adverse drug reaction after the instillation of cyclopentolate (1%) eyedrops was monitored. 

Statistical analysis

The continuous and categorical values were expressed as mean ± SD and percentage, respectively. The nonparametric tests (Kruskal-Wallis test) were used to make group comparisons. A Pearson correlation test was performed to identify the correlation between the variables, and a p-value of <0.05 was considered statistically significant. All analysis was performed using IBM SPSS Statistics for Windows, version 24.0 (IBM Corp., Armonk, New York, United States).

## Results

A total of 130 participants in the age group of 5-15 years were recruited for the study. Out of these, 20 participants, who could not achieve the desired pupillary dilatation (≥ 4 mm) at the end of one hour, were excluded, resulting in a final sample size of 110. The demographic characteristics are illustrated in Table 1.

**Table 1:** Demographic characteristics of the study population

Parameters	Values
Age (Years), mean±SD (range)	11.33 ± 3.23 (5-15)
Age Group, n (%)	
5- ≤10 Years	39 (35.5%)
>10-15 Years	71 (64.5%)
Gender, n (%)	
Male	48 (43.6%)
Female	62 (56.4%)
Visual acuity, n (%)	
6/6 - 6/12	90 (81.8%)
6/18-6/60	19 (17.3%)
Worse than 6/60	1 (0.9%)

Outcomes

**Figure 1:**
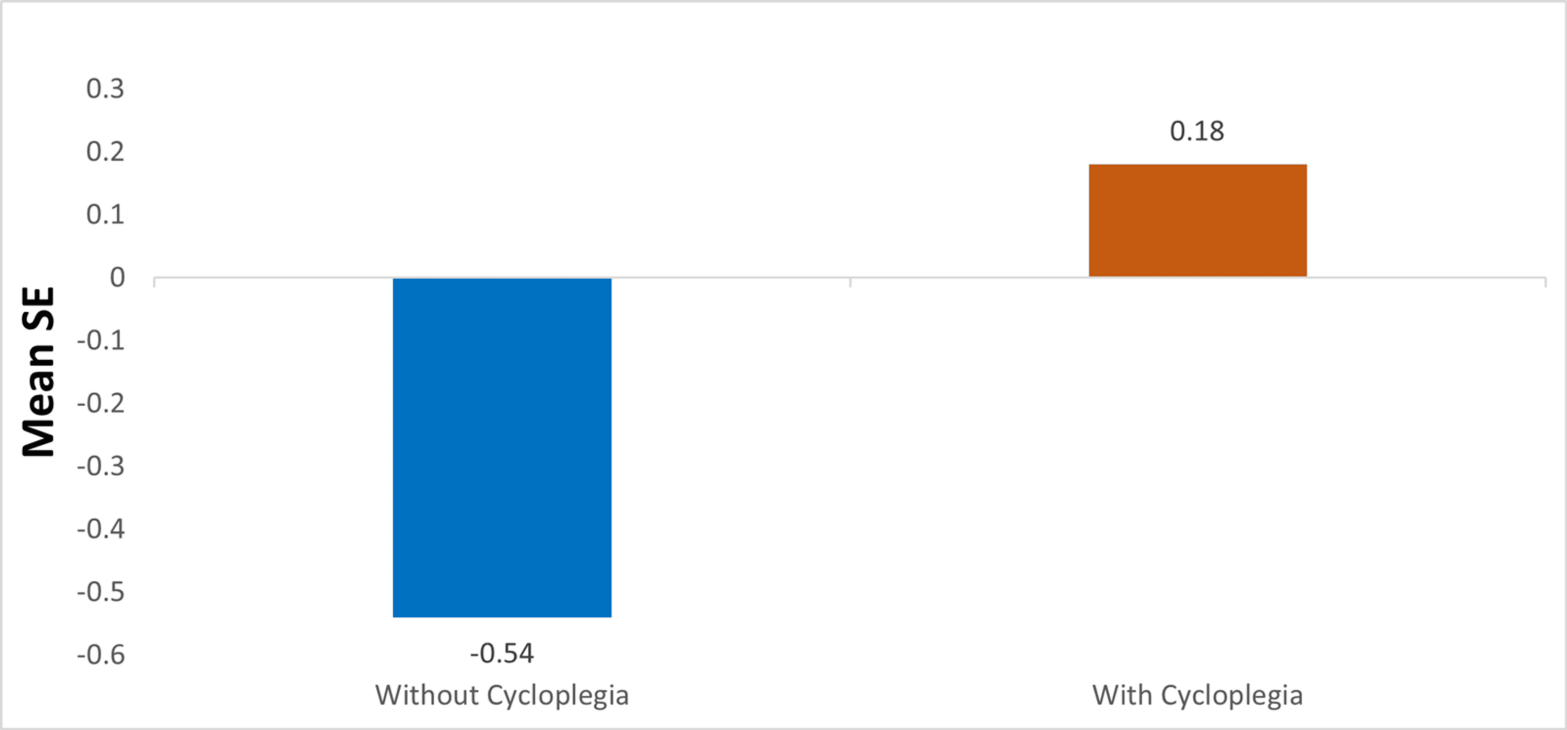
Comparison of mean spherical equivalent with and without cycloplegia. SE- spherical equivalent

The mean spherical equivalent (SE) (without cycloplegic) was -0.54, and the mean SE (with cycloplegic) was +0.18, as illustrated in Figure 1.

The mean ± SD of the difference between cycloplegic and non-cycloplegic refraction was 0.72 ± 0.72 diopter (D). The mean ± SD of the absolute difference of SE (without cycloplegic refraction) and SE (with cycloplegic refraction) for children with myopia, emmetropia, and hypermetropia has been shown in Tables 2, 3, respectively.

**Table 2:** Absolute difference in SE (without cycloplegia) in the three groups SE: spherical equivalent; SD: standard deviation; IQR: interquartile range Myopia is defined as SE < 0, Emmetropia is SE of 0; Hypermetropis is SE >0

Absolute Difference	SE (Without Cycloplegia)			Kruskal-Wallis Test	
	Children with myopia (n=69)	Children with emmetropia (n=6)	Children with hypermetropia (n=35)	Statistics	P-Value
Mean ± SD	0.44 ± 0.44	0.64 ± 0.28	0.87± 0.76	13.707	0.001
Median (IQR)	0.25 (0.125-0.56)	0.625 (0.375-0.75)	0.625 (0.375-1.25)		
Min -Max	0-1.75	0.375-1.125	0-3.25		

**Table 3:** Absolute difference in SE (with cycloplegia) in the three groups SE: spherical equivalent; SD: standard deviation; IQR: interquartile range Myopia is defined as SE < 0, Emmetropia is  SE of 0, Hypermetrope is  SE >0

Absolute Difference	SE (With Cycloplegia)			Kruskal-Wallis Test	
	Children with myopia (n=45)	Children with emmetropia (n=2)	Children with hypermetropia (n=63)	Statistics	p-value
Mean ± SD	0.40 ± 0.42	0.62 ± 0.71	0.73 ± 0.66	9.594	0.008
Median (IQR)	0.25 (0.12-0.5)	0.62 (0.38-0.88)	0.5 (0.25-1.06)		
Min - Max	0 - 1.75	0.12 - 1.12	0 - 3.25		

**Figure 2:**
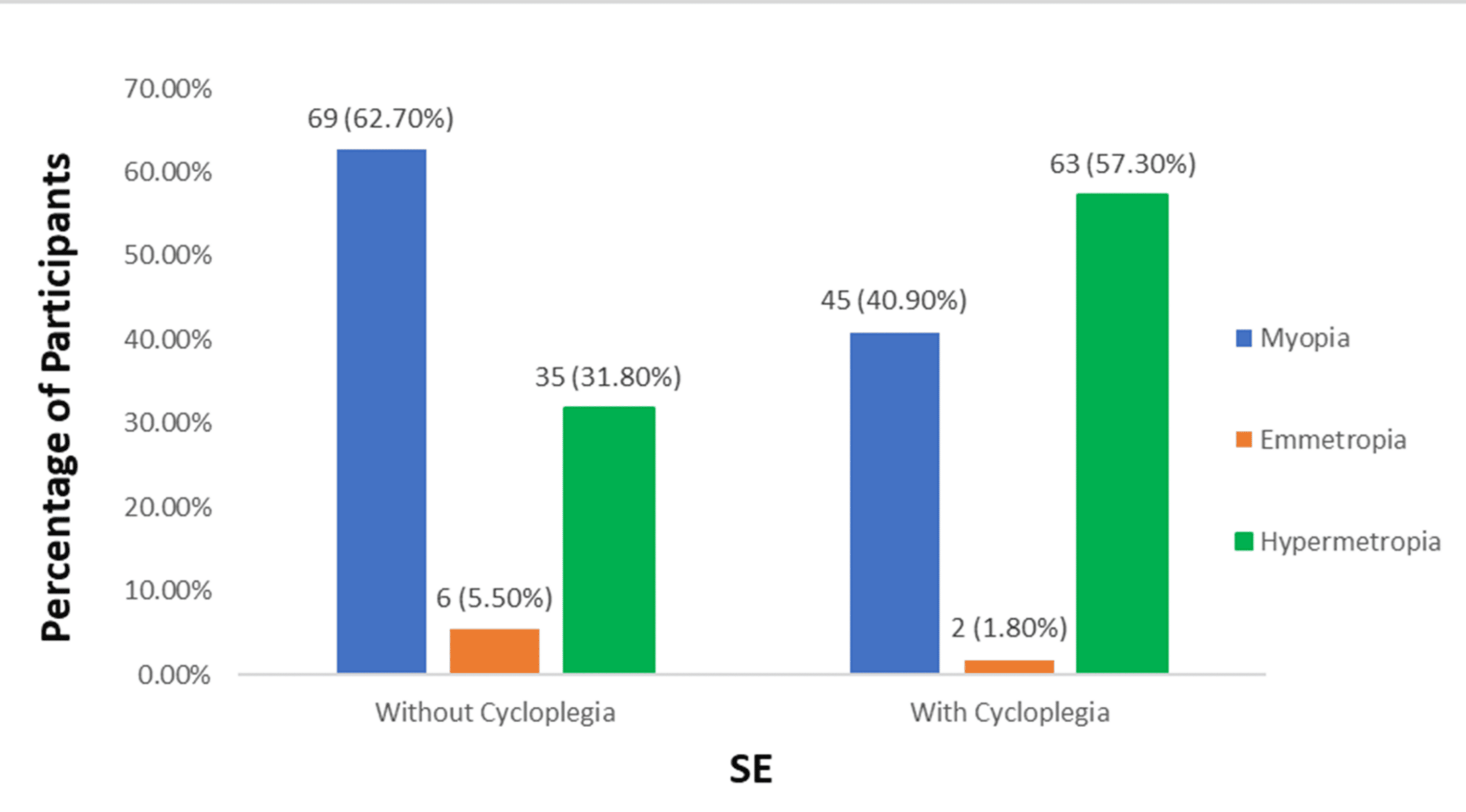
Distribution of participants (with and without cycloplegia) in terms of SE in the three groups (N=110) SE: spherical equivalent

 The mean Absolute difference of SE in the age group 5-10 years was 0.77, and that in the age group of 11-15 years was 0.48. The mean Absolute difference of SE for male children was 0.60, and that for female children was 0.57. The mean Absolute difference of SE in VA: 6/6-6/12 was 0.63, VA: 6/18-6/60 was 0.40, and <6/60 was 0.25. Figure 2 demonstrates the distribution of participants (with and without cycloplegia) in terms of SE in children with myopia, emmetropia, and hypermetropia.

Non-cycloplegic autorefraction results in the overestimation of myopia by 75.6% and underestimation of hypermetropia by 93.7%. The sensitivity and specificity for myopia were 100% and 63.1%, respectively, and for hyperopia, they were 55.6% and 100%, respectively. 

**Figure 3:**
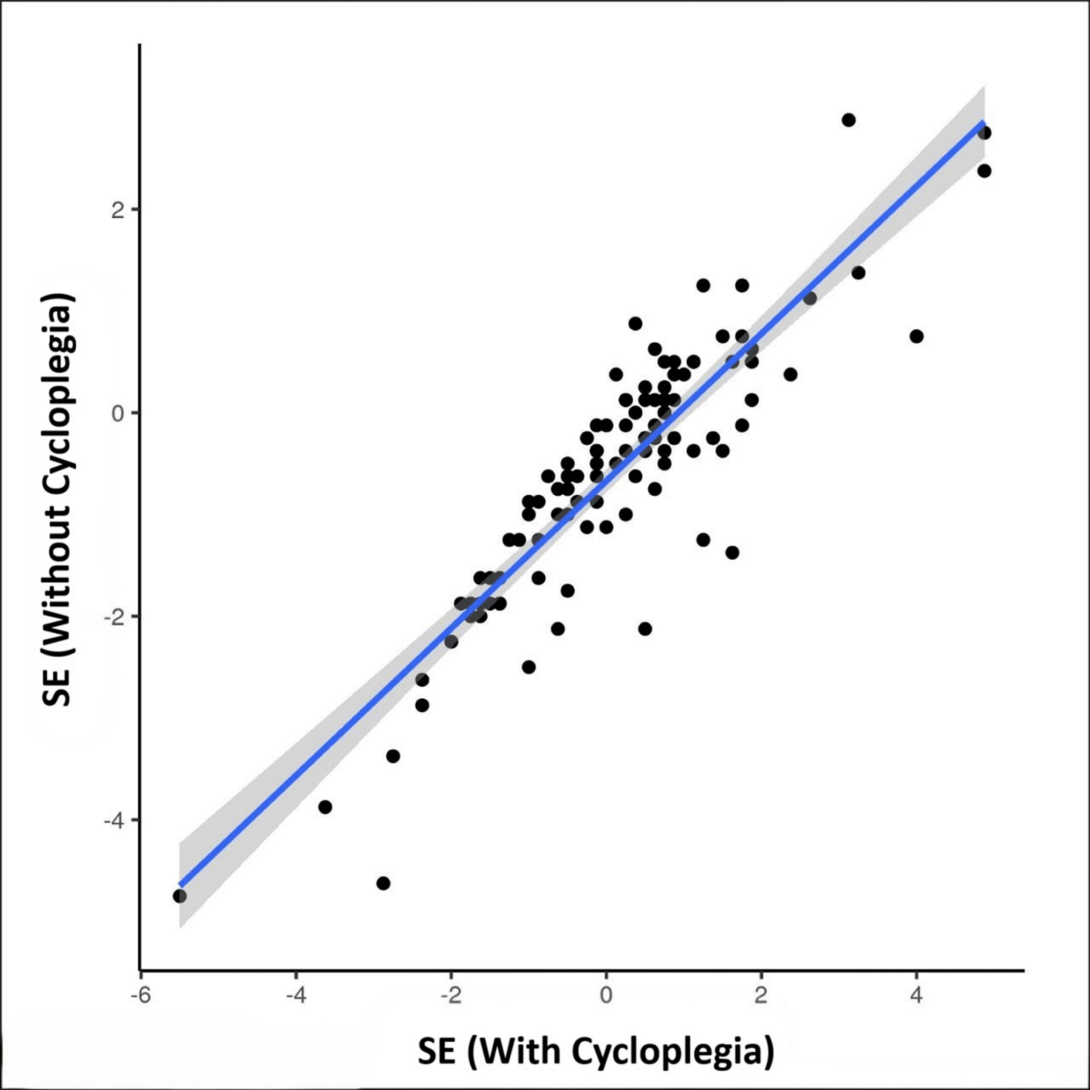
Correlation between SE (with cycloplegia) and SE (without cycloplegia). Pearson correlation test was performed to identify the correlation between the variables and was observed to be significant (correlation coefficient = 0.87, p = <0.001) Note: Individual points represent individual cases. The blue trendline represents the general trend of correlation between the two variables. The shaded grey area represents the 95% confidence interval of this trendline. SE: spherical equivalent

The scatterplot, as shown in Figure 3, depicts the correlation between SE (with cycloplegia) and SE (without cycloplegia). There was a strong correlation between SE (with cycloplegia) and SE (without cycloplegia), and this correlation was statistically significant (Interclass Correlation Coefficient = 0.87, p = <0.001).

Adverse drug reactions

No adverse drug reactions were documented in the study.

## Discussion

Over the past 20 years, the prevalence of myopia has significantly increased in India, rising from 4% in 2001 [11,12] to 13% in 2015 [13]. Moreover, it has also been reported that there is a three to six-fold rise in myopia post-home confinement due to the pandemic, among school children in India [14]. There are various limitations to using cycloplegia for refraction testing in children, and it can only be done in the presence of an ophthalmologist. Some of the obstacles that restrict the use of cycloplegics are the limited supply of cycloplegic drops, regulatory authorization for optometrists to use cycloplegics, and parental reluctance to submit their child to cycloplegics due to the extended effect of cycloplegic medications, causing blurred near vision. Consequently, under these circumstances, non-cycloplegic refraction is frequently utilized to ascertain the status of refractive error [15]. On the contrary, non-cycloplegic refractive error assessment leads to a more myopic measurement compared to cycloplegic assessment. As a result, it overestimates the occurrence of myopia and underestimates the prevalence of hypermetropia [16]. This has also been established in our study. Thus, this study emphasizes the need for cycloplegic refraction for the precise measurement of refractive error in children.

In the present study, non-cycloplegic autorefraction results in an overestimation of myopia by 75.6% and an underestimation of hypermetropia by 93.7%. Sankaridurg et al. found that non-cycloplegic autorefraction overestimated myopia by 28.5% and underestimated hypermetropia by 73% compared to cycloplegic autorefraction [17]. They also found that eyes with greater visual acuity, eyes with less myopic or more hyperopic refractive error, and younger age are factors that lead to overestimation of myopia using non-cycloplegic autorefraction [17]. They have defined myopia as ≤ -0.75 D and hyperopia ≥ +0.75 D, whereas in our study, no such cut-off values were used. This resulted in 40.6% of the values with SE (without cycloplegia) in children with myopia to be > -0.75. Similarly, 68.6% of values with SE (without cycloplegia) in children with hypermetropia were < +0.75. This could be the probable reason for the difference in percentages of overestimation of myopia and underestimation of hypermetropia.

The degrees of variation in refractive error before and after cycloplegic refraction may differ across various populations [8]. In our study, the mean ± SD of the difference between cycloplegic and non-cycloplegic refraction was 0.72 ± 0.72 D. Zhao et al. conducted a study including over 5,000 Chinese children of similar age and discovered that cycloplegic refraction was associated with a mean difference of 1.23 D higher hypermetropia or less myopia [18]. The Shandong Children Eye study found that among children aged 4-18 years, the difference between cycloplegic and non-cycloplegic refraction was 0.78 D [19]. A different study carried out in Australian schools by Fotedar et al., found that the mean spherical equivalent difference between these measures (post-cycloplegic refraction than pre-cycloplegic refraction) was 0.84 D in the 12-year-old children and 1.18 D in the six-year-old children [20]. The Sankara Nethralaya Tamil Nadu Essilor Myopia (STEM) study discovered a +0.34 D difference between cycloplegic and non-cycloplegic refraction [21]. Differences in participant variables, such as age and ethnicity, as well as methodological problems, including variations in refraction techniques, may be the cause of variations among studies [8]. The accommodating response to proximal cues during non-cycloplegia is probably what accounts for the dioptric discrepancy between cycloplegic and non-cycloplegic assessments [17].

In this study, the mean ± SD of the Absolute difference of SE (with cycloplegia) for children with myopia was 0.40 ± 0.42, for emmetropia was 0.62 ± 0.71, and for hypermetropia was 0.73 ± 0.66. This finding confirms previous research indicating that myopic eyes exhibit less accommodation for near targets, thereby minimizing the disparity between cycloplegic and non-cycloplegic refraction [22]. According to Guo et al, the mean SE difference for emmetropic, myopic, and hypermetropic eyes was -0.51 ± 0.83, -0.21 ± 0.89, and -1.14 ± 1.05, respectively (P<0.001). Across all age groups, myopic eyes showed the least variation in SE, with emmetropic eyes following closely after [16].

The mean Absolute difference of SE in the age group 5-10 years was 0.77, and that in the age group 11-15 years was 0.48. The mean Absolute difference of SE for male children was 0.60, and that for female children was 0.57. The mean Absolute difference of SE in VA 6/6-6/12 was 0.63, VA 6/18-6/60 was 0.40, and VA <6/60 was 0.25. According to Sankaridurg et al., younger age and hyperopic refractive error were associated with the largest mean paired difference in non-cycloplegic and cycloplegic refractive error. Additionally, the eyes with uncorrected VA poorer than or equivalent to 6/18 showed the least difference (0.26). Gender did not significantly affect the difference in refraction between cycloplegic and non-cycloplegic individuals [17]. According to Guo et al., the mean SE difference dropped (one-way analysis of variance, P<0.001) from -0.75 ± 1.82 in children under five years old to -0.47± 0.76 in individuals ≥ 15 years old [16]. The strength of the overall SE difference and the discovery that younger people were more likely to have a larger SE difference corroborate previous findings [17,23,24]. The age-related decline in accommodation may account for the tendency towards smaller disparities in SE with an increase in age. In their investigation, gender did not affect the overall SE disparities [16].

Sankaridurg et al. employed cut-off values of ≥+0.75 D for hypermetropia and ≤-0.75 D for myopia [17]. Guo et al. classified hypermetropia as having SE of at least +0.50 and myopia as having SE of at least -0.50 [16]. In the STEM study, hypermetropia was defined as a cycloplegic SE refraction of +2.00 or more [25]. Myopia was defined as having a SE objective refractive error of <-0.50 in the study by Flitcroft et al. [26]. The Shandong eye research classified hypermetropia as a refractive error of +0.75 D or more, myopia as a refractive error of at least -0.50 D, and emmetropia as a refractive error of >-0.50 D to <+0.75 D [19]. In our study, no such cut-offs were used. This has led to a variation in the prevalence of myopia and hypermetropia.

The study conducted by Sankaridurg et al. involving school-aged children in China by using cycloplegic refraction, showed that the prevalence of myopia, emmetropia, and hypermetropia was 28.7%, 28.2%, and 43.1%, respectively. The prevalence of myopia, emmetropia, and hypermetropia was estimated to be 36.9%, 51.7%, and 11.5% using non-cycloplegic refractive data, with only 62% of the eyes correctly categorized into their respective groups. The sensitivity was good in identifying myopia, but it was quite low in detecting hypermetropia, at 25.6% [17]. According to the Shandong eye study, 66.4% of eyes with non-cycloplegic refraction remained myopic, while 33.6% of eyes turned emmetropic (18.0%) or hypermetropic (15.7%) under cycloplegia. Before cycloplegia, the prevalence of emmetropia was 37.5%; after cycloplegia, it was 19.8%, and the remaining eyes were hypermetropic [19]. In the current study, the observed prevalence of myopia, emmetropia, and hypermetropia using cycloplegic refraction was 40.9%, 1.8%, and 57.3% respectively. Whereas without cycloplegia, 62.7% were myopic, 5.5% were emmetropic, and 31.8% were hypermetropic. In 74.5% of cases, the eyes were correctly classified in their respective categories. The sensitivity for myopia was 100% and that for hypermetropia was 55.6% with non-cycloplegic refraction, indicating that many hypermetropic eyes were incorrectly classified as either emmetropic or myopic. Therefore, if a child exhibits asthenopic symptoms, it is advised to refer them for cycloplegic refraction in a community setting to rule out latent hypermetropia and other binocular vision abnormalities [21].

Strengths and limitations

The strength of this study lies in its large sample size, high participation rate, and the collection of both pre- and post-cycloplegic autorefraction data. In this study, we utilized 1% cyclopentolate eyedrops, which is a commonly used cycloplegic agent in research related to refractive errors in children. It is effective even for moderate to high hypermetropia, with an onset of action approximately 30 minutes after administration and a duration of action of up to 24 hours. Furthermore, it is associated with fewer side effects compared to other cycloplegic agents [27]. None of the participants in this study reported any side effects related to the use of cyclopentolate. 

The present study aimed to distinguish cycloplegic from non-cycloplegic refraction. Therefore, we solely relied on autorefraction findings and did not conduct binocular subjective refraction. Moreover, doing subjective refraction can lead to interobserver bias. Also, we did not use subjective refraction as the endpoint for our comparison because we have included very young children in our study, whose subjective responses are less predictable than those of younger adults. We have used a single autorefractor (Topcon KR-1) to assess the difference between cycloplegic and non-cycloplegic refraction. The type of target and its size in an autorefractor are known to affect the accommodative response. Hence, results obtained without cycloplegia may differ from one instrument to another [28]. 

One limitation of the current study was that all participants who had achieved the pre-defined cut-off for pupillary dilatation at the end of one hour were taken up for cycloplegic refraction assessment. Thus, we are unable to ascertain whether cycloplegia was entirely established in each eye in the study. It is well known that the Asian population has lighter coloured iris as compared to the African population [29]. It has been claimed that iris pigment may sequestrate cycloplegic drugs, making it difficult to achieve complete cycloplegia in children with darker irises [8]. Since iris pigmentation varies greatly among ethnic groups and is mostly influenced by genetic ancestry, our results might not apply to other ethnic groups directly.

## Conclusions

In our study, myopia was overestimated and hypermetropia was underestimated with non-cycloplegic refraction as compared to cycloplegic refraction. Hence, despite being cumbersome and time-consuming, cycloplegia is still necessary to obtain the correct refractive error in children. Overall, this study provided valuable insights regarding the need for cycloplegic refraction in children, especially in light of the rising myopia trends, which have become a public health concern worldwide.

## References

[1] Kulkarni S, Gilbert C, Giri N, Hankare P, Dole K, Deshpande M (2022). Visual impairment and blindness among children from schools for the blind in Maharashtra state, India: changing trends over the last decade. Indian J Ophthalmol.

[2] Fricke TR, Holden BA, Wilson DA, Schlenther G, Naidoo KS, Resnikoff S, Frick KD (2012). Global cost of correcting vision impairment from uncorrected refractive error. Bull World Health Organ.

[3] (2024). World Health Organization: Blindness and vision impairment. https://www.who.int/news-room/fact-sheets/detail/blindness-and-visual-impairment.

[4] Tahhan N, Papas E, Fricke TR, Frick KD, Holden BA (2013). Utility and uncorrected refractive error. Ophthalmology.

[5] Sheeladevi S, Seelam B, Nukella PB, Modi A, Ali R, Keay L (2018). Prevalence of refractive errors in children in India: a systematic review. Clin Exp Optom.

[6] Flitcroft DI (2012). The complex interactions of retinal, optical and environmental factors in myopia aetiology. Prog Retin Eye Res.

[7] Farhood QK (2012). Cycloplegic refraction in children with cyclopentolate versus atropine. J Clin Exp Ophthalmol.

[8] Zhu D, Wang Y, Yang X (2016). Pre- and post-cycloplegic refractions in children and adolescents. PLoS One.

[9] Sharma S, Shah JS (2020). A comparative study of noncycloplegic refractive error values with cycloplegic refractive error values using autorefractometer. Int J Med Ophthalmol.

[10] Morgan IG, Iribarren R, Fotouhi A, Grzybowski A (2015). Cycloplegic refraction is the gold standard for epidemiological studies. Acta Ophthalmol.

[11] Murthy GV, Gupta SK, Ellwein LB (2002). Refractive error in children in an urban population in New Delhi. Invest Ophthalmol Vis Sci.

[12] Dandona R, Dandona L, Srinivas M (2002). Refractive error in children in a rural population in India. Invest Ophthalmol Vis Sci.

[13] Saxena R, Vashist P, Tandon R, Pandey RM, Bhardawaj A, Menon V, Mani K (2015). Prevalence of myopia and its risk factors in urban school children in Delhi: the North India Myopia Study (NIM Study). PLoS One.

[14] Saara K, Swetha S, Subhiksha R, Amirthaa M, Anuradha N (2022). Steep increase in myopia among public school-going children in South India after COVID-19 home confinement. Indian J Ophthalmol.

[15] Williams C, Miller L, Northstone K, Sparrow JM (2008). The use of non-cycloplegic autorefraction data in general studies of children's development. Br J Ophthalmol.

[16] Guo X, Shakarchi AF, Block SS, Friedman DS, Repka MX, Collins ME (2022). Noncycloplegic compared with cycloplegic refraction in a Chicago school-aged population. Ophthalmology.

[17] Sankaridurg P, He X, Naduvilath T (2017). Comparison of noncycloplegic and cycloplegic autorefraction in categorizing refractive error data in children. Acta Ophthalmol.

[18] Zhao J, Mao J, Luo R, Li F, Pokharel GP, Ellwein LB (2004). Accuracy of noncycloplegic autorefraction in school-age children in China. Optom Vis Sci.

[19] Hu YY, Wu JF, Lu TL (2015). Effect of cycloplegia on the refractive status of children: the Shandong children eye study. PLoS One.

[20] Fotedar R, Rochtchina E, Morgan I, Wang JJ, Mitchell P, Rose KA (2007). Necessity of cycloplegia for assessing refractive error in 12-year-old children: a population-based study. Am J Ophthalmol.

[21] Gopalakrishnan A, Hussaindeen JR, Sivaraman V (2021). The Sankara Nethralaya Tamil Nadu Essilor Myopia (STEM) study-defining a threshold for non-cycloplegic myopia prevalence in children. J Clin Med.

[22] Millodot M (2015). The effect of refractive error on the accommodative response gradient: a summary and update. Ophthalmic Physiol Opt.

[23] Fotouhi A, Morgan IG, Iribarren R, Khabazkhoob M, Hashemi H (2012). Validity of noncycloplegic refraction in the assessment of refractive errors: the Tehran Eye Study. Acta Ophthalmol.

[24] Li T, Zhou X, Zhu J, Tang X, Gu X (2019). Effect of cycloplegia on the measurement of refractive error in Chinese children. Clin Exp Optom.

[25] Negrel AD, Maul E, Pokharel GP (2000). Refractive error study in children: sampling and measurement methods for a multi-country survey. Am J Ophthalmol.

[26] Flitcroft DI, He M, Jonas JB (2019). IMI - Defining and classifying myopia: a proposed set of standards for clinical and epidemiologic studies. Invest Ophthalmol Vis Sci.

[27] Major E, Dutson T, Moshirfar M (2020). Cycloplegia in children: an optometrist's perspective. Clin Optom (Auckl).

[28] Suryakumar R, Bobier WR (2003). The manifestation of noncycloplegic refractive state in pre-school children is dependent on autorefractor design. Optom Vis Sci.

[29] Albert DM, Green WR, Zimbric ML, Lo C, Gangnon RE, Hope KL, Gleiser J (2003). Iris melanocyte numbers in Asian, African American, and Caucasian irides. Trans Am Ophthalmol Soc.

